# Supplementary material for: Aromatase Inhibitors and Risk of Arthritis and Carpal Tunnel Syndrome among Taiwanese Women with Breast Cancer: A Nationwide Claims Data Analysis
Source: J Clin Med. 2020 Feb 19;9(2):566. doi: 10.3390/jcm9020566 (PMC7074454; doi:10.3390/jcm9020566)
Supplement: Supplementary file 1 [file jcm-09-00566-s001.pdf]

Supplementary Materials: Aromatase Inhibitors and Risk of Arthritis and Carpal Tunnel Syndrome among Taiwanese Women with Breast Cancer: A Nationwide Claims Data Analysis

Hsu-Chih Chien, Yea-Huei Kao Yang *, C. Kent Kwoh, Pavani Chalasani, Debbie L. Wilson and Wei-Hsuan Lo-Ciganic *

Table S1. Operational Definitions of Medications.

Medications (by Therapeutic Class)	ATC Code(s)
Anti-cancer therapy	
Anastrozole	L02BG03
Anthracyclines	L01DB
Exemestane	L02BG06
Chemotherapy	L01
Letrozole	L02BG04
Tamoxifen	L02BA01
Taxanes	L01CD
Analgesics	
Acetaminophen	M03B
Gabapentinoids	N03AX12, N03AX16
Nonsteroidal anti-inflammatory drugs	N02AJ, N02B
Opioids	N02A
Diabetes	
Alpha-glucosidase inhibitors	A10BF
Biguanides	A10BA, A10BD01, A10BD02, A10BD03, A10BD05, A10BD07, A10BD08, A10BD10, A10BD11, A10BD13, A10BD14, A10BD15
Dipeptidyl peptidase 4 inhibitors	A10BH, A10BD07, A10BD08, A10BD09, A10BD10, A10BD11, A10BD12, A10BD13, A10BD18
Sulfonylureas	A10BB, A10BC, A10BD01, A10BD02, A10BD04, A10BD06, A10BD14
Insulins	A10A
Thiazolidinediones	A10BG, A10BD03, A10BD04, A10BD05, A10BD06, A10BD09, A10BD12
Hypertension	
Angiotensin converting enzyme inhibitors	C09A, C09B
Angiotensin receptor blockers	C09C, C09D
Beta blockers	C07
Calcium channel blockers	C08C, C08D, C08E, C08G, C09BB, C09Db, C09XA53 and C09XA54
Diuretics	C02L, C03, C07B, C07C, C07D, C08G, C09BA, C09DA, C09XA52 and C09XA54
Dyslipidemia	
Bile acid sequestrants	C10AC
Ezetimibe	C10AX09, C10BA02, C10BA05, C10BA06
Fibrates	C10AB, C10BA03, C10BA04
Niacins	C10AD, C10BA01
Statins	C10AA, C10BA, C10BX
Other medications	

Anticoagulants	B01AA
Antidepressants	N06A
Antiplatelets	B01AC, C10BX01, C10BX02, C10BX04, C10BX05, C10BX06, C10BX08
Hypnotic benzodiazepines	N05CD
Non-benzodiazepam hypnotics	N05CF
Pamidronic acid (intravenous)	M05BA03
Thyroxine	H03AA
Zoledronic acid (intravenous)	M05BA08

Table 2. Operational Definitions of Disease and Procedures[a].

**Diseases (Ordered Alphabetically)**	**Diagnosis (ICD-9 Diagnose Codes)**
Anxiety disorders	300x
Any arthritis	714.0x, 714.1x, 714.2x, 714.30, 714.31, 714.32, 714.33, 715.00, 715.04, 715.09, 715.10, 715.11, 715.12, 715.13, 715.14, 715.15, 715.16, 715.17, 715.18, 715.20, 715.21, 715.22, 715.23, 715.24, 715.25, 715.26, 715.27, 715.28, 715.30, 715.31, 715.32, 715.33, 715.34, 715.35, 715.36, 715.37, 715.38, 715.80, 715.89, 715.90, 715.91, 715.92, 715.93, 715.94, 715.95, 715.96, 715.97, 715.98, 719.xx, 720.0x, 721.0x, 721.1x, 721.2x, 721.3x, 721.90, 721.91
Bone metastasis	198.5x
Breast cancer	174.x
Carpal tunnel syndrome	354.0x
Chronic kidney disease	585.xx
Coronary arterial diseases	410.x, 411.x, 412.x, 413.x, 414.x (excluding 414.1x)
Depressive disorders	311.x
Diabetes mellitus	250.x
Dyslipidemia	272.x
Hypertension	401.x-409.x (excluding 402.11, 402.9)
Liver cirrhosis	571.2x, 571.3x, 571.5x and 571.6x
Other non-breast cancers	140.x-239.x (excluding 174.x)
Wrist fracture	813.x, 814.x, 815.x 817.xx
Procedures (including image workups)	ICD-9 procedure codes or other local coding system
Bone scan	26029B, 26075B (local coding system)
Computed tomography	33070B, 33071B, 33072B (local coding system)
Magnetic resonance imaging	33084B, 33085B (local coding system)
Positron emission tomography	26072B, 26073B (local coding system)
Primary tumor resection	85.2x, 85.4x
Wrist surgery	77.x4, 78.x4, 80.x3

[a]Women were considered to have the comorbidities and/or use the medication of our interests if there were at least two medical claims during the year before endocrine therapy initiation.

Table S3. Propensity-Scored Matched Analyses: Variables Included and Matching Algorithms.

	Any Arthritis		Carpal Tunnel Syndrome	
**Matching Algorithms with Variables Included**	**Letrozole vs. Anastrozole**	**Taxane-Based vs. Taxane-Free Chemotherapy**	**Letrozole vs. Anastrozole**	**Taxane-Based vs. Taxane-Free Chemotherapy**
Year of endocrine therapy initiation	+	+	+	+
Primary tumor resection	+		+	
Propensity score				
Common variables[a]	+	+	+	+
Primary tumor resection		+		+
Chemotherapy	+		+	
Taxanes	+		+	
Endocrine therapy		+		+
Wrist fracture			+	+
Thyroxine			+	+

Abbreviations: PS: propensity score. +: included. [a] Common variables in the propensity score models are: age, comorbidities (including anxiety disorders, chronic kidney disease, coronary arterial diseases, depressive disorders, diabetes mellitus, dyslipidemia, hypertension and liver cirrhosis), medications (including alpha-glucosidase inhibitors, angiotensin converting enzyme inhibitors, angiotensin receptor blockers, anticoagulants, antiplatelets, beta blockers, biguanides, bile acid sequestrants, calcium channel blockers, dipeptidyl peptidase 4 inhibitors, diuretics, dyslipidemia, ezetimibe, fibrates, gabapentinoids, benzodiazepine hypnotics, insulins, niacins, non-benzodiazepine hypnotics, nonsteroidal anti-inflammatory drugs/acetaminophen, opioids, statins, sulfonylureas and thiazolidinediones) and treatments and image workups related to breast cancer (including use of anthracyclines, receiving bone scan, computed tomography, magnetic resonance imaging and positron emission tomography).

Table S4. Aromatase Inhibitors Use and Risk of Any Arthritis and Carpal Tunnel Syndrome: Multivariable Cause-specific Cox model (Full Model).

	Any Arthritis	Carpal Tunnel Syndrome
	**Multivariable Analysis[a] (aHR, 95% CI)**	**Multivariable Analysis[a] (aHR, 95% CI)**
**Types of Endocrine Therapy (Ref: Tamoxifen)**		
Anastrozole	1.11 (0.94–1.31)	1.78 (1.07–2.95)
Exemestane	1.10 (0.74–1.63)	1.32 (0.32–5.38)
Letrozole	1.27 (1.12–1.44)	1.66 (1.13–2.43)
Age (years of age, Ref: less than 45)		
45 to 54	1.63 (1.44–1.85)	1.78 (1.31–2.44)
55 to 64	2.31 (2.03–2.64)	1.17 (0.80–1.71)
65 to 74	2.91 (2.50–3.38)	0.52 (0.29–0.96)
More than 75	3.18 (2.65–3.81)	0.15 (0.04–0.64)
Year of endocrine therapy initiation (Ref: before 2010)		
After 2011	0.98 (0.90–1.05)	1.22 (0.97–1.54)
History of treatment for breast cancer[b]		
Primary tumor resection	0.98 (0.87–1.09)	0.98 (0.69–1.41)
Radiation therapy	1.16 (1.05–1.27)	1.18 (0.90–1.55)
Chemotherapy (Ref: non-taxane-based)		
No chemotherapy	1.00 (0.91–1.10)	0.97 (0.73–1.27)

Taxane-based	0.92 (0.81–1.05)	0.97 (0.67–1.40)
NCI index (Ref: NCI index=0)		
1	1.11 (1.00–1.24)	1.17 (0.83–1.65)
≥2	1.12 (0.95–1.33)	0.78 (0.39–1.55)
Comorbidities/ Medications[c]		
Hypertension	1.11 (1.02–1.21)	1.08 (0.83–1.41)
Diabetes	0.95 (0.83–1.08)	0.71 (0.43–1.16)
Dyslipidemia	1.18 (1.06–1.30)	1.31 (0.93–1.85)
Affective disorders	1.32 (1.23–1.43)	1.34 (1.06–1.70)
Chronic kidney disease	0.74 (0.51–1.07)	1.36 (0.40–4.65)
Liver cirrhosis	1.06 (0.70–1.59)	0.62 (0.09–4.44)
Wrist fracture	-	4.02 (1.65–9.80)
Opioids	1.37 (1.19–1.59)	1.38 (0.90–2.13)
NSAIDs/acetaminophen	1.32 (1.19–1.47)	1.28 (0.92–1.78)
Thyroxine	-	1.06 (0.47–2.38)
Type of treatment hospitals		
Capital area	0.84 (0.78–0.91)	0.87 (0.69–1.09)
Medical center	1.06 (0.99–1.15)	0.80 (0.64–1.00)

Abbreviations: aHR: adjust hazard ratio; CI: confidence interval; Ref: reference [a] Multivariable cause-specific Cox models for the risk of any arthritis and carpal tunnel syndrome both adjusted for age, year of initiating endocrine therapy, history of primary tumor resection, radiation therapy, chemotherapy, National Cancer Institute index, history of hypertension, diabetes, dyslipidemia, affective disorders, chronic kidney disease, liver cirrhosis, use of opioids, non-steroidal anti-inflammatory drugs/acetaminophen, hospital location, and hospital type. The model for carpal tunnel syndrome additionally adjusted for history of wrist fracture and use of thyroxine. [b] Prior treatment was measured within 12 months before endocrine therapy initiation. [c] Hypertension, diabetes, dyslipidemia and affective disorders were identified by ICD-9 codes and/or medications within 12 months before endocrine therapy initiation. The remaining were identified by ICD-9 codes.

Table S5. Risk of Any Arthritis and Carpal Tunnel Syndrome: Multivariable Cause-specific Cox Models Using Propensity Scored Cohorts (Sensitivity Analyses).

**Outcomes**	**Letrozole Compared to Anastrozole (aHR, 95% CI)[a]**	**Taxane vs. Non-Taxane-Based Chemotherapy (aHR, 95% CI)[a]**
Any arthritis	1.07 (0.82–1.39)	0.87 (0.72–1.04)
Carpal Tunnel Syndrome	0.77 (0.34–1.75)	1.00 (0.60–1.66)

Abbreviations: aHR: adjust hazard ratio; CI: confidence interval; PS: propensity score [a] Multivariable cause-specific Cox models for the risk of any arthritis and carpal tunnel syndrome both adjusted for age, year of initiating endocrine therapy, history of primary tumor resection, radiation therapy, chemotherapy, National Cancer Institute index, history of hypertension, diabetes, dyslipidemia, affective disorders, chronic kidney disease, liver cirrhosis, use of opioids, non-steroidal anti-inflammatory drugs/acetaminophen, hospital location, and hospital type. The model for carpal tunnel syndrome additionally adjusted for history of wrist fracture and use of thyroxine.
